# The relationship between time spent during the first ANC contact, home visits and adherence to ANC contacts in Ghana

**DOI:** 10.1080/16549716.2021.1956754

**Published:** 2021-08-17

**Authors:** Maxwell Tii Kumbeni, Paschal Awingura Apanga, Eugene Osei Yeboah, Jonas Timbire Kolog, Baba Awuni

**Affiliations:** a Ghana Health Service, Nabdam District Health Directorate, Nangodi, Ghana; b School of Public Health, University of Nevada, Reno, Reno, USA; c Ghana Health Service, Bolgatanga East District Health Directorate, Zuarungu, Ghana

**Keywords:** Eight or more anc contacts, time spent during first ANC contact, home visits, pregnant women, ghana

## Abstract

**Background:**

The World Health Organization in 2016 recommended eight or more antenatal care (ANC) contacts for a positive pregnancy experience, however, it is unclear what impact the time spent during the first ANC contact and home visits can have on eight or more ANC contacts.

**Objectives:**

Our study investigated the relationship between time spent during the first ANC contact and eight or more ANC contacts, and between home visits and eight or more ANC contacts. We also assessed the prevalence of eight or more ANC contacts.

**Methods:**

A cross-sectional study was conducted among 519 mothers with children 12 months old and below attending child welfare clinics in the Nabdam district in the Upper East Region, Ghana. Multivariable logistic regression analysis was used to assess the relationship between time spent during the first ANC contact, home visits, and eight or more ANC contacts, while controlling for potential confounders.

**Results:**

The proportion of mothers who attained eight or more ANC contacts during pregnancy was 31.2%. Spending 20 minutes or more during the first ANC contact was associated with 2.07 times the odds of having eight or more ANC contacts compared to spending less than 20 minutes [adjusted odds ratio (aOR): 2.07, 95% CI: 1.18,3.63]. Mothers who received at least a home visit from skilled health professionals during pregnancy were 2.44 times more likely to have eight or more ANC contacts compared to mothers who were not visited (aOR: 2.44, 95% CI: 1.51,3.94).

**Conclusion:**

Spending at least 20 minutes during the first ANC contact and home visits were positively associated with eight or more ANC contacts. We recommend that skilled health professionals should spend at least 20 minutes during the first ANC contact as well as encourage home visits in order to increase the coverage of eight or more ANC contacts.

## Background

Antenatal care (ANC) is an important strategy for reducing maternal morbidity and mortality through early identification and adequate management of high-risk pregnancies [1–3]. Regular monitoring of pregnant women during ANC by skilled health professionals enables high-risk pregnancies to be detected, reduce birth-related complications and promote safer motherhood [4,5]. Because of this, the World Health Organization (WHO) in 2016 reviewed the Focused Antenatal Care (FANC) model, which has a recommended minimum of four visits to a more expanded model with a recommended minimum of eight contacts. The expanded model requires pregnant women to make their first contact in the first trimester followed by two and five contacts in the second and third trimesters respectively [5]. Besides the recommended number of ANC contacts, the model emphasizes on the timing of each contact and the content of each encounter should be adaptable to the local context. The WHO posits that recent evidence found that the FANC model was associated with more perinatal deaths compared to the model with a minimum of eight contacts [5]. The organization also reported that irrespective of the resource setting, more ANC contacts are associated with maternal satisfaction compared to fewer contacts.

Apart from meeting the minimum recommended eight ANC contacts, adhering to the number of contacts per trimester plays an important role in delivering public health interventions during pregnancy. These interventions include tetanus vaccination, intermittent preventive treatment (IPTp) for malaria, and prevention of mother-child-transmission (PMTCT) of HIV [6]. However, many low-and-middle-income countries (LMIC) are neither meeting the recommended number of eight ANC contacts nor adhering to the number of contacts per trimester of pregnancy [7,8]. A recent study in Benin reported that 8% of pregnant women had eight or more ANC contacts with the healthcare provider [9], while other studies in Bangladesh and Myanmar reported that only 6% and 18% of pregnant women respectively, had eight or more ANC contacts [10,11].

The first ANC contact is an important point to establish good rapport between the pregnant woman and the skilled health professional [12]. It allows the skilled health professional to have an interaction with the pregnant woman and to assess the pregnancy [13]. The assessment of the pregnancy may include history taking, physical examination, laboratory investigations, which are essential to identify high-risk pregnancies. It is also the time to discuss ANC appointments and birth preparation [13]. Therefore, the first ANC contact requires adequate time to do a comprehensive evaluation of the pregnant woman unlike subsequent contacts [14]. However, it is not known how the time spent with skilled health professionals during the first ANC contact might affect pregnant women on meeting the 2016 WHO’s ANC contact recommendations.

Home visits during pregnancy provide healthcare to pregnant women and are associated with improved maternal health and birth outcomes [15–17]. However, there is a paucity of information on home visits by skilled health professionals to pregnant women during ANC [16,18], and these findings are mixed. While a non-randomized population-based intervention in a fragile country such as Afghanistan by Edmond et al. reported that home visits to pregnant women by trained community health workers was positively associated with the increased number of ANC contacts compared to pregnant women who were not visited [18], a cluster randomized controlled trial in Nigeria by Cockcroft et al. found a null association between home visits to pregnant women and eight or more ANC contacts [16].

The government of Ghana introduced the free maternal healthcare policy in 2008 to improve the utilization of ANC, skilled delivery, and postnatal care service in the country [19,20]. The 2017 Ghana Maternal Health Survey report revealed that approximately 98% and 90% of pregnant women in Ghana had at least one ANC contact and four or more ANCs contacts respectively, with skilled health professionals [21]. However, it is not known how many of the pregnant women had at least eight ANC contacts according to the 2016 WHO’s ANC recommendations. It is also not known how time spent during first ANC contact and home visits to pregnant women might impact on ANC utilization in Ghana. The primary aims of our study were to assess the relationship between time spent during the first ANC contact and eight or more ANC contacts, and between home visits to pregnant women and eight or more ANC contacts. Our secondary aim was to assess the proportion of pregnant women who adhered to the 2016 WHO’s recommendations on eight or more ANC contacts among pregnant women in Nabdam district in the Upper East Region, Ghana.

## Methods

### Health profile of study setting

Nabdam district is in the Upper East region of Ghana. It has an estimated population of 41,646 out of which 9,995 are women of reproductive age. A total of 970 women gave birth in 2020. Among the deliveries, 973 were live births whiles 9 were still births. All the pregnant women had at least one ANC contact [22]. Approximately one-third of pregnant women in the Nabdam district are nulliparous. The healthcare facilities in the district include four health centers, two clinics, and 18 Community-based Health Planning and Services (CHPS) Compounds. An estimated 100 community health nurses and midwives provide ANC services across all the healthcare facilities in the district [22].

### Study design and population

A cross-sectional study was conducted between April to May 2021. The study population was mothers attending child welfare clinics in Nabdam district.

### Inclusion and exclusion criteria

Mothers aged 18 years old or above who delivered a live birth in the past 12 months and attended antenatal care while they were pregnant with their current child in the Nabdam district were eligible for inclusion in the study. Mothers without their maternal and child health record book or mothers who attended ANC elsewhere outside of the district were excluded from the study. The maternal and child health record book is an integrated booklet that has records of all stages of maternal, newborn, and child health; from ANC to delivery, postnatal care, child vaccination, and child growth monitoring [23]. It therefore has data on when the woman was pregnant for the live birth within the past 12 months.

### Data collection tools

Face-to-face interviews were conducted with the women using close-ended questionnaires. Data were also collected by reviewing the maternal and child health record books.

### Sample size

Epi Info Version 7.1 (STAT CALC) was used to estimate the sample size for the study. As the prevalence of ANC eight contacts was not known, a 50% prevalence rate was used with a 95% confidence interval and a 5% margin of error. The estimated sample size was 407 including a 10% non-response rate. However, 519 mothers participated in the study.

### Sampling procedure

All child welfare clinics were purposively selected for the study, and mothers who met the inclusion criteria were conveniently sampled. Data were collected from mothers at the premises of the selected child welfare clinics according to their postnatal care scheduled dates.

### Outcome variable

The outcome variable of interest was eight or more ANC contacts to align with the 2016 WHO’s recommendations. Eight or more ANC contacts was defined as ‘having at least eight ANC contacts; one in the first trimester, two in the second trimester, and five in the third trimester when the mother was pregnant [5].’ We extracted the number of ANC contacts per trimester from the maternal and child health record book to generate our outcome variable. This was coded dichotomously as ≥8 contacts versus 0–7 contacts.

### Predictor variables

We had two predictor variables of interest in our study. The predictor variables of interest were time spent during the first ANC contact and home visits to the mother when she was pregnant. Time spent during the first ANC contact was defined as ‘the number of minutes spent with a skilled health professional during the first ANC contact excluding waiting time,’ and was categorized as ≥20 minutes and <20 minutes. We compared mothers who spent at least 20 minutes during the first ANC contact to mothers who spent less than 20 minutes during the first ANC contact when they were pregnant. We used a time distribution plot from the self-reported data on time spent during the first ANC contact to guide the categorization of time in our study.

Home visits were defined as ‘any routine or special visits by a skilled health professional to assess the home of the pregnant woman and to provide counseling and any other care that may be required.’ Mothers who received any home visit were also compared to mothers who did not receive such a visit when they were pregnant.

### Covariates

The covariates in our study were socio-demographic and obstetric-related variables. The socio-demographic covariates were age, marital status, mother’s education, employed, partner’s education, place of residence, and health insurance status. Age was categorized as 18–22 years, 23–27 years, ≥28; marital status (married, single); mother’s education (no formal education, primary, secondary, higher); employed (yes, no); partner’s education (no formal education, primary, secondary, higher); place of residence (rural, urban); and health insurance status (yes, no).

Obstetric-related covariates included planned pregnancy, parity, payment for any ANC contact, chronic disease in pregnancy, and did COVID-19 pandemic interfere with your ANC attendance. These were categorized as follows; planned pregnancy (yes, no); parity (0–3, ≥4); payment for any ANC contact (yes, no); chronic disease in pregnancy (yes, no); and did COVID-19 pandemic interfere with your ANC attendance (yes, no). Payment for any ANC contact was any monetary payment that was made by the mother during ANC regardless of her health insurance status. Chronic disease was defined as any of the following diseases reported during pregnancy; hypertension, diabetes, asthma, cardiac disease, chronic kidney disease, sickle cell disease, mental illness, tuberculosis and thyroid disease.

The variables age, marital status, mother’s education, employed, partner’s education, place of residence, parity, number of ANC contacts, and chronic disease in pregnancy were extracted from the maternal and child health record book, whiles insurance status, payment for any ANC contact, planned pregnancy, home visits, time spent during the first ANC contact and whether COVID-19 interfered with my ANC attendance were self-reported during the survey. The variable selection was guided by previous literature [9,10,24].

### Data analysis

Proportions and frequencies were used to present data on the characteristics of study participants. Bivariate and multivariable logistic regression analyses were used to assess the relationship between the predictor variables and outcome variable, while adjusting for potential confounders (i.e. all covariates). Potential confounders were specified as *a priori* as we thought that there was a biological plausibility that they are associated with the predictor variables and outcome of interest. Model fitness was determined using the global null hypothesis test. We also assessed the model for issues of multicollinearity to ensure that this was not a problem. Statistical significance was considered at P-value <0.05.

We conducted a sensitivity analysis to assess the association between time spent during the first ANC contact and eight or more ANC contacts to see whether there was a dose-response relationship between time spent during the first ANC contact and eight or more ANC contacts. We compared the prevalence of eight or more ANC contacts among mothers who spent 20–40 minutes during the first ANC contact, and mothers who spent greater than 40 minutes during the first ANC contact, to mothers who spent less than 20 minutes during the first ANC as the reference category. SAS version 9.4 (SAS Institute, Cary NC) was used for the data analysis.

## Results

### Characteristics of the study sample

A total of 519 mothers participated in the study. Most mothers were aged 18–22 years (40.8%), and 86.9% of mothers were married. More than half (54.8%) of the mothers had primary education, while 37.2% of their partners had primary education. Most of the mothers (65.5%) were from rural areas and 62.8% had health insurance.

About one-third (31.2%) of the mothers had eight or more ANC contacts and all mothers had at least one ANC contact. An estimated 13.3% of mothers had a parity of four or more. Mothers spent a mean time of 33 minutes ± 20.6 during their first ANC contact. A quarter of the mothers (26.1%) were visited at home by a skilled health professional during their pregnancy. Majority of the mothers (80.7%) did not make any payment during ANC contacts and 94.4% of the mother’s reported that the COVID-19 pandemic did not interfere with their ANC attendance (Table 1).

**Table 1.** Characteristics of the study sample

Variable	N (%) or Mean (SD)
*Socio-demographic characteristics*	
Mother’s age (years)	
18–22	212 (40.8)
23–27	119 (22.9)
≥ 28	188 (36.2)
Marital status	
Married	451 (86.9)
Single	68 (13.1)
Mother’s education	
No formal education	120 (23.2)
Primary	284 (54.8)
Secondary	91 (17.6)
Higher	23 (4.4)
Employed	127 (24.5)
Partner’s education	
No formal education	178 (34.5)
Primary education	192 (37.2)
Secondary	90 (17.4)
Higher	56 (10.9)
Place of residence	
Rural	340 (65.5)
Urban	179 (34.5)
Has health insurance	326 (62.8)
*Obstetric-related characteristics*	
Antenatal care (ANC) contacts	
0–7	357 (68.8)
≥ 8	162 (31.2)
Planned pregnancy	344 (66.4)
Parity	
0–3	450 (86.7)
≥4	69 (13.3)
Time spent at first ANC contact (Minutes)	33 (20.6)
Home visits	135 (26.1)
Payment for any ANC contact	100 (19.3)
Has chronic disease in pregnancy	28 (5.4)
COVID-19 pandemic interfered with my ANC attendance?	29 (5.6)

### The association between time spent during the first ANC contact, home visits, and eight or more ANC contacts

The results show that spending 20 minutes or more during the first ANC contact was associated with 2.07 times the odds of having eight or more ANC contacts compared to spending less than 20 minutes [adjusted odds ratio (aOR): 2.07, 95% CI: 1.18,3.63]. Mothers who were visited at home during pregnancy were also 2.44 times more likely to have eight or more ANC contacts compared to those who were not visited (aOR: 2.44, 95% CI: 1.51,3.94).

We also found that the odds of having eight or more ANC contacts was 6.02 times among mothers with higher education compared to those with no formal education (aOR: 6.02, 95% CI: 1.49,24.3). Mothers whose pregnancies were planned had 2.44 times the odds of having eight or more ANC contacts compared to those whose pregnancies were unplanned (aOR: 2.44, 95% CI: 1.46,4.08). For mothers who reported that their ANC attendance was interfered by the COVID-19 pandemic, they had 77% lower odds of attaining eight or more ANC contacts (aOR: 0.23, 95 CI: 0.06,0.85) (Table 2).

**Table 2.** The association between time spent at first ANC contact, home visits, and antenatal care

Variable	Unadjusted OR (95% CI)	**Adjusted OR (95% CI)
Time spent at firstANCcontact		
< 20 minutes	1	1
≥ 20 minutes	1.32 (0.81,2.13)	2.07 (1.18,3.63) *
Home visits		
No	1	1
Yes	2.20 (1.46,3.32)	2.44 (1.51,3.94) *
Mother’s age (years)		
18–22	1	1
23–27	1.63 (1.01,2.65)	1.25 (0.72,2.18)
≥ 28	1.37 (0.89,2.11)	1.50 (0.83,2.71)
Marital status		
Married	1	1
Single	0.59 (0.32,1.08)	1.00 (0.48,2.08)
Mother’s education		
No formal education	1	1
Primary education	1.39 (0.85,2.27)	1.62 (0.89,2.95)
Secondary	1.62 (0.89,2.97)	2.24 (1.00,5.01)
Higher	5.88 (2.25,15.37)	6.02 (1.49,24.3) *
Employed		
No	1	1
Yes	1.48 (0.97,2.26)	0.91 (0.53,1.55)
Partner’s education		
No formal education	1	1
Primary education	1.18 (0.75,1.86)	0.97 (0.56,1.68)
Secondary	1.46 (0.84,2.54)	1.10 (0.56,2.15)
Higher	2.60 (1.39,4.85)	1.04 (0.43,2.52)
Place of residence		
Rural	1	1
Urban	1.43 (0.97,2.10)	1.35 (0.87,2.11)
Health insurance status		
No	1	1
Yes	1.50 (1.01,2.23)	1.13 (0.71,1.80)
Planned pregnancy		
No	1	1
Yes	2.44 (1.58,3.77)	2.44 (1.46,4.08) *
Parity		
0–3	1	1
≥4	0.57 (0.31,1.05)	0.60 (0.27,1.30)
Payment for any ANC contact		
No	1	1
Yes	1.11 (0.69,1.77)	1.47 (0.87,2.49)
Chronic disease in pregnancy		
No	1	1
Yes	0.87 (0.38,2.03)	0.77 (0.27,2.20)
COVID-19 pandemic interfered with my ANC attendance?		
No	1	1
Yes	0.24 (0.07,0.81)	0.23 (0.06,0.85) *

OR = odds ratio; * = Significant at P – value < 0.05; 1 = Reference category; **Adjusted model included all covariates.

Our sensitivity analysis to see whether there was a dose-response relationship between time spent during the first ANC contact and eight or more ANC contacts found no dose-response relationship. We observed that mothers who spent 20–40 minutes during the first ANC contact had 2.23 times the odds of attaining eight or more ANC contacts compared to mothers who spent less than 20 minutes during the first ANC contact (aOR:2.23, 95% CI: 1.14,4.37). In addition, the prevalence of eight or more ANC contacts among mothers who spent greater than 40 minutes was 2.01 times the odds of mothers who spent less than 20 minutes during the first ANC contact (aOR:2.01, 95% CI: 1.12, 3.59). The sensitivity analysis showed similar results to that observed in our main analysis.

## Discussion

The primary aims of this study were to investigate the relationship between time spent during the first ANC contact and eight or more ANC contacts, and between home visits to pregnant women and eight or more ANC contacts. We also assessed the proportion of pregnant women who adhered to the WHO’s recommendation of eight or more ANC contacts. We found that mothers who spent at least 20 minutes with a skilled health professional during the first ANC contact were likely to attain eight or more ANC contacts compared to mothers who spent less than 20 minutes. In addition, attaining eight or more contacts was more prevalent among mothers who were visited at home during pregnancy by a skilled health professional compared to mothers who were not visited. The proportion of mothers who attained eight or more ANC contacts during pregnancy was 31.2%.

The findings of our study indicate that mothers who spent 20 minutes or more with a skilled health professional during the first ANC contact were more likely to attain eight or more ANC contacts. The first ANC contact is an essential component of ANC and usually requires history taking, physical examination as well as laboratory investigations [13]. During this period, high-risk pregnancies can be identified and managed appropriately. It is also during the first ANC contact that subsequent appointments can be discussed with the pregnant women [13]. The first ANC contact, therefore, requires adequate time for skilled health professionals to conduct physical examination, laboratory investigations and to have discussions with the pregnant woman comprehensively [14]. Spending at least 20 minutes with each woman might be adequate for the skilled health professional to carry out all that is needed during the first ANC contact and might be the reason the odds of attaining eight or more ANC contacts doubled among such women. Since the average time spent by mothers during the first ANC contact in our study was 33 minutes, we propose that if skilled health professionals can spend at least 20 minutes with each pregnant woman during the first ANC contact, the coverage of the eight or more ANC contacts recommended by WHO might be doubled in this setting.

Our study also found higher odds of attaining eight or more ANC contacts among mothers who were visited at home during pregnancy by skilled health professionals. Home visits provide skilled health professionals the opportunity to assess the pregnant woman at home and offer counsel as well as deliver any care that may be needed [25]. Home visits can also build trust between the skilled health professional and the pregnant woman, which we believe is essential in disseminating healthcare information. Our finding corroborates with findings by a recent study by Edmond et al. in Afghanistan [18], but was inconsistent with findings of Cockcroft et al. in Nigeria who reported a null relationship between home visits to pregnant women and the number of ANC contacts [16]. While trained healthcare workers were involved in home visits in the current study in Ghana and in that of Afghanistan, the home visits in the study in Nigeria were not provided by trained healthcare workers. This might have accounted for the discrepancy in our findings as trained health workers are more skilled in healthcare counseling and caregiving.

The prevalence of eight or more ANC contacts according to our study was 31.2% among mothers in the Nabdam district of Ghana. This prevalence is higher than previous findings from Benin (8%), Bangladesh (6%), and Myanmar (18%) [9–11]. The higher prevalence observed in our study be due to the free maternal healthcare policy in Ghana [19]. This policy is said to have removed financial barriers directly related to the utilization of maternal and child health services in Ghana [26]. Meanwhile, a recent study conducted by Ekholuenetale et al. in Ghana reported a prevalence of 41.9% for eight or more ANC contacts among women of reproductive age [27]. While the assessment of eight or more ANC contacts in our study aligned well with the 2016 WHO’s recommendations, Ekholuenetale and his colleagues assessed the number of ANC contacts regardless of the timing of each contact within the trimester of pregnancy.

We also found that mothers with higher education were more likely to attain eight or more ANC contacts compared to mothers with no formal education. Pregnant women with higher education might be exposed and informed about maternal and child health, and are more likely to take a proactive approach to adequate utilization of ANC services [28]. Furthermore, higher education is associated with living in urban areas where healthcare facilities are readily available and this may also lead to adequate utilization of ANC services [29]. Our analysis also showed that mothers who reported planned pregnancy had higher odds of attaining eight or more ANC contacts compared to mothers with an unplanned pregnancy. This finding aligns with previous studies [30,31]. We also observed that mothers who did not access ANC because of the COVID-19 pandemic had lower odds of attaining eight or more ANC contacts. This is not surprising because the fear of contracting the virus could have scared pregnant women from adhering to their routine ANC schedules.

This study had some strengths and limitations. Our findings are not generalizable because of the convenient sample for our study. Nevertheless, our study is the first of its kind to assess the relationship between time spent during the first ANC contact and eight or more ANC contacts. It is also the first study in Ghana to assess the association between home visits to pregnant women and eight or more ANC contacts. We did have a high response rate and missingness in our data was not a problem. Some of the variables in our study including time spent during the first ANC contact were self-reported and may be subject to recall bias, but this should not differ between mothers who had eight or more ANC contacts and mother who did not. We do not also expect recall bias on our primary outcome of interest as this was assessed using the maternal and child health record book, which records the timing and the number of ANC contacts made with the healthcare facility when she was pregnant. Our study also used a cross-sectional design which cannot establish causal relationships.

## Conclusion

Approximately one-third of the mothers in our study received eight or more ANC contacts. Spending at least 20 minutes during the first ANC contact and home visits to pregnant women by skilled health professionals were associated with meeting the 2016 WHO’s recommended eight or more ANC contacts. To improve the coverage of ANC contacts, we recommend that skilled health professionals spend at least 20 minutes during the first ANC contact and home visits should also be encouraged.

## Author contributions

MTK, PAA, EOY, JTK, and BA conceptualized and designed the study. MTK, EOY, JTK, and BA contributed to data collection. MTK and PAA analyzed the data. MTK, PAA, EOY, JTK, and BA wrote the initial manuscript. All authors reviewed and approved the final manuscript.

## Supplementary Material

Supplemental Material

## Paper context

It is not known whether time spent during first antenatal care contact is associated with eight or more contacts. There is also paucity of research on the relationship between home visits to pregnant women and eight or more antenatal care contacts. Therefore, we assessed the relationship between time spent during the first antenatal care contact and eight or more antenatal care contacts, and between home visits to pregnant women and eight or more antenatal contacts.
