# Supplementary material for: The relationship between time spent during the first ANC contact, home visits and adherence to ANC contacts in Ghana
Source: Glob Health Action. 2021 Aug 17;14(1):1956754. doi: 10.1080/16549716.2021.1956754 (PMC8381959; doi:10.1080/16549716.2021.1956754)
Supplement: Supplemental Material [file ZGHA_A_1956754_SM5116.docx]

**Study Questionnaire**

**Socio-demographic characteristics**

1. How old were you when you were pregnant with this child? ……………..years
2. Were you married when you were pregnant with this child?
3. Never Married [ ]
4. Married [ ]
5. Divorced/Widowed [ ]
6. Educational level when your pregnant with this child:
7. No formal education [ ]
8. Primary [ ]
9. Secondary [ ]
10. Tertiary [ ]
11. Where was your place of residence when you were pregnant with this child?
12. Rural
13. Urban
14. While you were pregnant were you employed?
15. Yes [ ]
16. No [ ]
17. At the time you registered for ANC at the clinic did you have a valid health insurance?
18. Yes [ ]
19. No [ ]
20. Were you ever asked to pay money for any service during your ANC?
21. Yes [ ]
22. No [ ]
23. How much time in your estimation would you say was spent with the skilled health professional at your first ANC visit (excluding waiting time)? …………………………….(minutes)
24. What is the level of education of your partner (husband or boyfriend)?
25. No formal education [ ]
26. Primary [ ]
27. Secondary [ ]
28. Tertiary [ ]

**Obstetric and gynecological characteristics**

1. Parity (excluding this child) …………………………
2. Did you plan for the pregnancy of this child?
3. Yes [ ]
4. No [ ]
5. Number of ANC contacts for each trimester:

P1. First trimester ……………….

P2. Second trimester ……………

P3. Third trimester ……………….

1. While you were pregnant with this child were you ever visited at home by any health worker at any time within your pregnancy?
2. Yes [ ]
3. No [ ]
4. Did you have any chronic disease (hypertension, diabetes, asthma, cardiac disease, chronic kidney disease, sickle cell disease, mental illness, tuberculosis and thyroid disease.) while you were pregnant with this child?
5. Yes [ ]
6. No [ ]
7. Did the COVID-19 pandemic deter you from attending ANC while you were pregnant with this child?
8. Yes [ ]
9. No [ ]

**THANK YOU!!!!!!!!**
